# Efficacy and safety of endoscopic submucosal dissection for papillary adenocarcinoma-type early gastric cancer

**DOI:** 10.1097/MD.0000000000016134

**Published:** 2019-06-21

**Authors:** Jae Ho Park, Ju Seok Kim, Sun Hyung Kang, Hee Seok Moon, Jae Kyu Sung, Hyun Yong Jeong

**Affiliations:** Departments of Internal Medicine, Chungnam National University School of Medicine, Daejeon, Korea.

**Keywords:** early gastric cancer, endoscopic submucosal dissection, papillary adenocarcinoma

## Abstract

Endoscopic submucosal dissection (ESD) has increasingly been used to treat early gastric cancer (EGC); however, its efficacy in treating papillary adenocarcinoma-type EGC remains unknown.

We sought to identify risk factors for lymph node (LN) metastasis in papillary adenocarcinoma-type EGC and evaluate the clinical outcome after ESD.

This study retrospectively reviewed the medical records of patients who were diagnosed with EGC in our hospital from January 2009 to December 2016. In total, 85 patients had papillary adenocarcinoma-type EGC, of whom 52 and 33 underwent surgical treatment and ESD, respectively. This study analyzed the LN metastasis risk factors and clinical outcomes between these 2 groups and with those of an existing ESD indication group.

LN metastasis occurred in 13 (25.0%) of 52 patients who underwent surgery. Multivariate analysis indicated that lymphovascular invasion was an independent risk factor (odds ratio: 20.624; 95% confidence interval: 19.628–21.497; *P* = .001). Of 33 patients who underwent ESD, 21 (63.6%) had an absolute indication and 12 (36.4%) had an expanded indication. All 3 (9.1%) patients with non-curative resection underwent additional surgery. The clinical outcomes were compared to those of 926 patients who underwent ESD of non-papillary adenocarcinoma-type EGC. There were no significant differences in curative resection rate (*P* = .327), procedure-related complication (*P* = .853), local recurrence (*P* = 1.000), or overall survival (*P* = 1.000).

ESD of papillary adenocarcinoma-type EGC showed an acceptable outcome in comparison to an existing ESD indication group. However, these patients exhibit a relatively higher risk of LN metastasis.

## 1 Introduction

Historically, early gastric cancer (EGC) has been treated primarily by surgery; however, endoscopic submucosal dissection (ESD) has been used with increasing frequency. ESD can help improve a patient's quality of life, reduce other morbidities associated with surgery, and preserve the stomach. Currently, it is used as a primary treatment option only if indicated.^[1,2]^ Recently, with improvements in endoscopic technique and instrumentation, ESD has been performed for expanded indications as well as under existing absolute indications. In studies using expanded indications, few complications related to the procedure have been reported and favorable long-term outcomes have been observed.^[3–5]^ In addition, ESD for EGC should be considered since the incidence of lymph node (LN) metastasis, which is of great concern for ESD, is not of particular importance for EGC.^[6]^ However, these studies are mainly concerned with well- to moderately-differentiated-type adenocarcinoma. Moreover, few studies exist using other histologic types of EGC, such as papillary adenocarcinoma.

Papillary adenocarcinoma is 1 histologic type of gastric adenocarcinoma whose characteristic pathology includes papillary epithelial processes and a thin fibrous core.^[7]^ Since it is a rare histologic type of gastric cancer and accounts for approximately 6% to 11% of all gastric cancers, very little is known regarding its biological characteristics or clinical progress.^[8]^ Papillary adenocarcinoma is classified as an intestinal-type under the Lauren classification, whereas it is considered as a differentiated-type under Japanese classification.^[9,10]^ However, it is known that it has a poor prognosis since it has a higher risk of LN metastasis and peritoneal metastasis in comparison to differentiated-type adenocarcinoma.^[7,11]^ For this reason, it is controversial whether the existing ESD indication should apply to papillary adenocarcinoma-type EGC, which shows a unique clinical progress and prognosis in comparison to other differentiated-types of adenocarcinoma.

Thus, this study sought to identify find risk factors for LN metastasis, which is of considerable concern for ESD, in papillary adenocarcinoma-type EGC patients. We further sought to analyze the efficacy and safety of ESD in papillary adenocarcinoma-type EGC and compared clinical outcomes with those of existing well- to moderately-differentiated adenocarcinoma-type EGC after ESD.

## 2 Methods

### 2.1 Study populations

This study retrospectively analyzed the medical records of patients who had been pathologically diagnosed with EGC in Chungnam National University Hospital (Daejeon, Korea) and had undergone surgery or ESD from January 2009 through December 2016. Patients who refused treatment or were transferred to another hospital after the EGC diagnosis, and those whom our hospital did not continuously follow up for more than 1 year after treatment, were excluded from the study. Patient demographics were compared between the surgery and ESD groups according to the treatment method. Risk factors were analyzed according to LN metastasis. ESD patients were further divided into a papillary adenocarcinoma group and a well- to moderately-differentiated adenocarcinoma group according to the pathological result. The clinical outcomes were compared between these 2 groups. For clinical staging before treatment, upper gastrointestinal (GI) endoscopy, abdominal computed tomography (CT), and chest radiography were conducted in all patients. EGC was classified as either elevated, flat, or depressed types according to macroscopic morphology and classified into upper third, middle third, or lower third of the stomach according to the location. We do not need an institutional review board approve because this is a retrospective study and has no impact on the patient.

### 2.2 Surgery

According to the tumor location, size, and macroscopically determined EGC type, total or subtotal gastrectomy was performed. For curative resection, at least 3 cm was left as a resection margin from the tumor and extended D2-lymphadenectomy was performed in most patients. To assess invasion into surrounding organs, *en bloc* resection was conducted and the resected specimen was examined according to the standard protocol. For all patients who underwent surgery, pathologic tumor, LN, and metastasis stage were reset according to the final pathological result.

### 2.3 ESD procedure

ESD was conducted by expert GI endoscopists. During the procedure, intravenous injections of the sedatives midazolam (Roche Korea Co., Ltd, Seoul, South Korea) and propofol were administered under cardio-pulmonary function monitoring. The drug capacity was controlled according to the degree of sedation. During the endoscopy, indigo-carmine solution was applied to clear up the boundary of the lesion before being marked with argon plasma coagulation. In addition, saline solution containing epinephrine and indigo carmine was injection and the lesion was dissected with an insulation-tipped (IT) diathermy knife or IT knife-2 (Olympus Medical, Tokyo, Japan). During the ESD procedure, high-frequency generators (ICC200 or VIO 300D; ERBE Elektromedizin, Tubingen, Germany) were used. A patient was considered to have experienced procedure-related complications, such as bleeding, if an additional endoscopic hemostatic method was necessary or if there were symptoms, such as melena or hematemesis. Perforation was defined as the detection of free air via chest or abdominal radiography after the procedure.

### 2.4 Pathologic examination

After the resected tissue was fixed in 10% formalin, serial sectioning was conducted at 2 mm intervals. Sections were then observed via microscopy. If there were more than 2 types of tumor cells, they were histologically classified according to the Japanese classification of gastric carcinoma based on the cell type that occupied more than 50% of the space.^[10]^ Tumor size, depth of invasion, whether there was a tumor cell in the resection margin, lymphovascular invasion (LVI), and LN metastasis were reviewed by experienced GI pathologists. Curative resection was defined as a case in which there were absent tumor cells in the lateral and basal resection margins and no LVI. Piecemeal resected tissue, as well as *en bloc* resected lesion, was defined as curative resection if the lateral and basal margins could be sufficiently identified after the reconstruction.

### 2.5 Follow-up

Complete blood cell count and chest and abdominal radiography were performed after ESD to check if any procedure-related complications occurred. If it was confirmed that there were no complications, a soft diet was begun on the day after the procedure. If the pathologic result indicated non-curative resection additional surgery was performed emergently. In cases of curative resection, GI endoscopy was performed at 3, 6, and 12 months after the procedure and annually thereafter. All lesions with a suspicious abnormality were assessed using endoscopic biopsy. Every 6 months, abdominal CT and chest radiography were performed. This interval was adjusted according to the condition or symptoms of each patient.

### 2.6 Statistical analysis

In order to compare categorical variables, such as clinical outcomes after ESD, a Chi-squared test or Fisher exact test was conducted. The categorical variables were indicated with frequencies and percentage while continuous variables were indicated with the mean and standard deviation (SD). For multivariate analysis, some variables, including depth of invasion and LVI, were adjusted and the odds ratio (OR) and confidence interval (CI) were reported. All of the above-described tests were 2-sided and *P* <.05 was considered to indicate significance. All statistical analyses were conducted using SPSS version 18.0 (SPSS Inc., Chicago, IL).

## 3 Results

### 3.1 Patients and clinical characteristics

**Figure 1:**
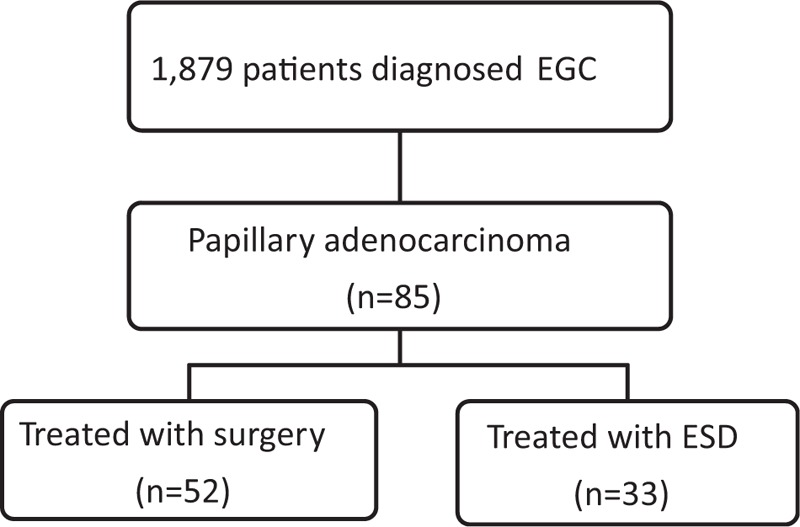
Flow chart of this study. EGC = early gastric cancer, ESD = endoscopic submucosal dissection.

Of 1879 patients who were diagnosed with EGC in our hospital during the research period; excluding 6 papillary adenocarcinoma patients who were not followed up for more than 1 year, 85 (4.5%) had papillary adenocarcinoma, of whom 59 (69.4%) were male and the average (standard deviation [SD]) age was 69.2 (9.1) years (Table 1). Among them, 52 patients (61.2%) underwent surgery whereas the remaining 33 (48.8%) underwent ESD (Fig. 1). Macroscopically, most cases were elevated (52.9%) and located in the lower third of the stomach (50.6%). The average (SD) of the tumor size was 24.8 (13.7) mm. Tumorous tissue was confined to the mucosa in 44 patients (51.8%), whereas 41 patients (48.2%) exhibited submucosal invasion. Among all patients, 33 (38.8%) had LVI. Moreover, in the surgery group, 13 patients (25.0%) exhibited LN metastasis. During the research period, 926 patients were diagnosed with well- to moderately-differentiated adenocarcinoma-type EGC and underwent ESD in our hospital as well as follow-up for more than 1 year.

**Table 1:**
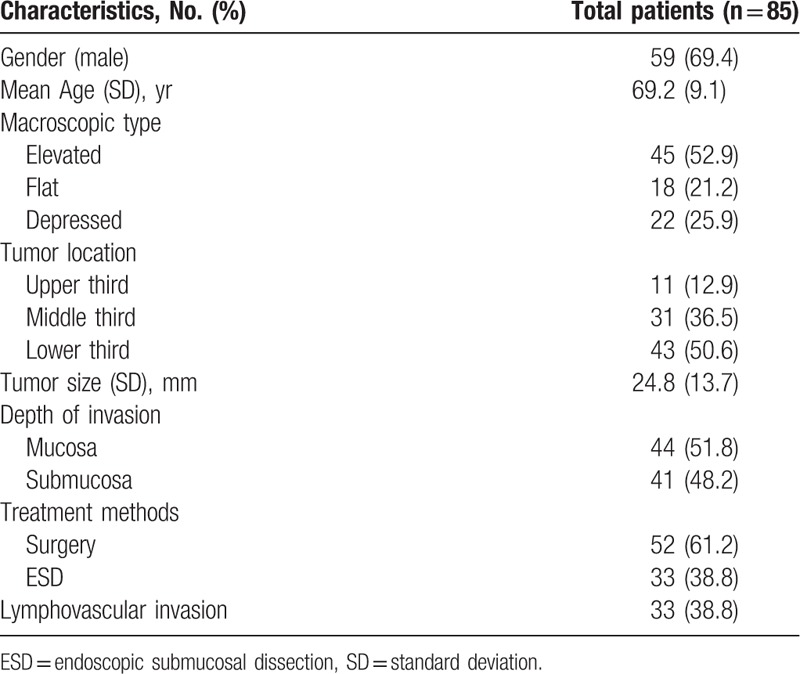
Baseline characteristics of patients with diagnosed papillary adenocarcinoma type early gastric cancer.

### 3.2 Risk factor of LN metastasis

The pathological reports of 52 patients who underwent surgery for papillary adenocarcinoma-type EGC were analyzed according to LN metastasis. In total, 13 patients (25.0%) exhibited LN metastasis. Their risk factors are reported in Table 2. Among the 52 surgical patients, 40 (76.9%) were male and the average (SD) age was 68.4 (10.2) years. Macroscopically, most were elevated (55.8%) and located in the lower third of the stomach (51.9%). EGC lesions were confined to the mucosa in 17 patients (32.7%) and 35 (67.3%) exhibited submucosal invasion. There were no differences in gender (*P* = .658), age (*P* = .502), macroscopic type (*P* = .165), tumor location (*P* = .821), LN enlargement on CT (*P* = .108), or tumor size (*P* = .541) between patients with and without LN metastasis. However, submucosal invasion of the tumor (*P* = .039) and LVI (*P* = .002) were statistically significantly higher in the group with LN metastasis. In order to identify independent risk factors for LN metastasis, the depth of invasion and LVI were adjusted to conduct a multivariate analysis. This analysis indicated that LVI (OR: 20.624; 95% CI: 19.628–21.497; *P* = .001) was the only risk factor of LN metastasis and that there was no significant difference in the depth of invasion (OR: 3.829; 95% CI: 0.854–9.511; *P* = .089) between the 2 groups.

**Table 2:**
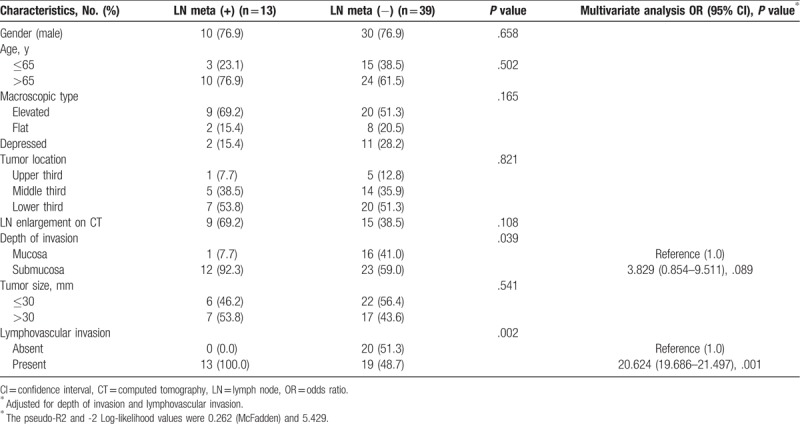
Risk factors of lymph node metastasis in patients with diagnosed papillary adenocarcinoma type early gastric cancer.

### 3.3 Clinical outcomes of ESD

**Figure 2:**
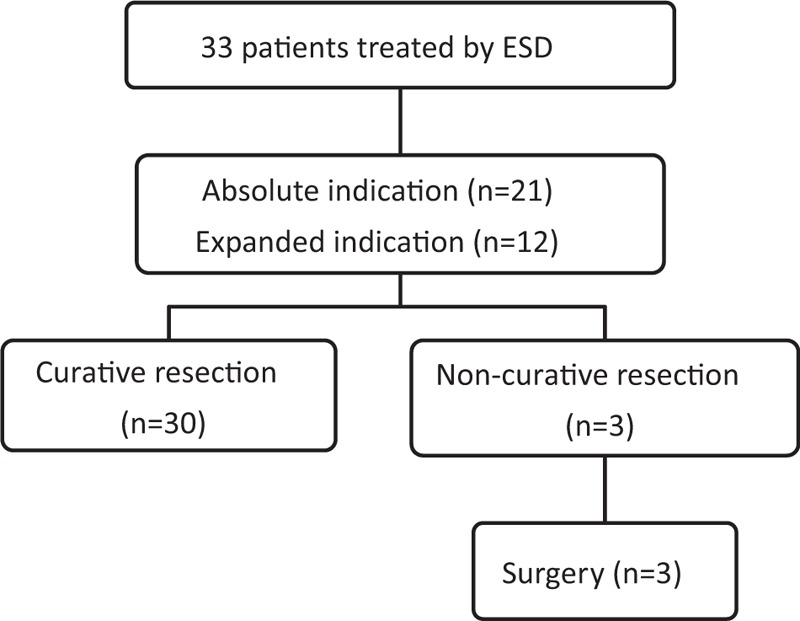
Endoscopic submucosal dissection results of papillary adenocarcinoma type early gastric cancer. ESD = endoscopic submucosal dissection.

There were 33 patients who underwent ESD with papillary adenocarcinoma-type EGC. We classified all patients according to the existing ESD guideline. In total, 21 patients had an absolute indication (intramucosal cancer without ulceration and size ≤20 mm); 12 patients had an expanded indication (intramucosal cancer without ulceration regardless of size and minute submucosal cancer in size ≤30 mm), and no patient had any above expanded indication. In the final pathological result, 30 patients (90.9%) exhibited curative resection, whereas the 3 patients who exhibited non-curative resection (9.1%), including 2 resection margin-positive patients and 1 person with LVI, underwent additional surgery. There was no LN metastasis in the pathological results after surgery (Fig. 2).

During the study period, 926 patients underwent ESD with well- to moderately-differentiated adenocarcinoma-type EGC. We compared their baseline characteristics and clinical outcomes after ESD with 33 patients who underwent ESD with papillary adenocarcinoma-type EGC (Table 3). Of the 959 patients from both groups, there were 640 men (66.7%) with an average (SD) age of 67.1 (8.9) years. Meanwhile, 19 patients (57.6%) in the papillary adenocarcinoma-type EGC group were male and 22 patients (66.7%) were over 65 years of age. Macroscopically elevated type was observed in 14 patients (42.4%) and most lesions were located in the lower third of the stomach. Most lesions were confined to the mucosa (84.4%) and LVI was observed in 1 patient (3.0%). In 2 patients (6.1%), tumor cells were observed in the resection margin and procedure-related complications occurred in 3 patients (9.1%). There was neither local recurrence nor patients who died during the observation period.

**Table 3:**
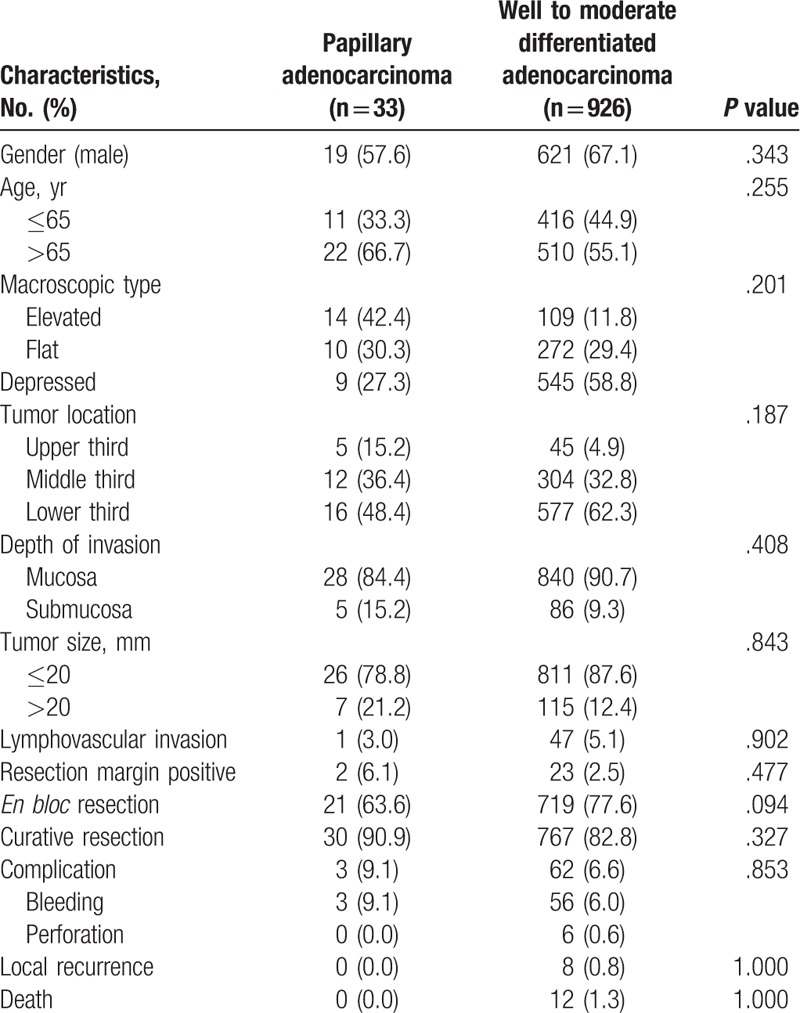
Compare of endoscopic submucosal dissection outcome in papillary adenocarcinoma and well to moderate differentiated adenocarcinoma type early gastric cancer.

We compared the well- to moderately-differentiated adenocarcinoma group and papillary adenocarcinoma group. In doing so, we observed no significant differences in baseline characteristics, including gender (*P* = .343), age (*P* = .255), macroscopic type (*P* = .201), tumor location (*P* = .187), depth of invasion (*P* = .408), or tumor size (*P* = .843). In addition, there were no significant differences in clinical outcomes after ESD, LVI (*P* = .902), resection margin positive rate (*P* = .477), *en bloc* resection rate (*P* = .094), curative resection rate (*P* = .327), procedure-related complications (*P* = .853), local recurrence (*P* = 1.000), or overall survival (*P* = 1.000) between the 2 groups. The average (SD) observation period of the papillary adenocarcinoma group was 39.5 (13.2) months and that of the well- to moderately-differentiated adenocarcinoma group was 37.7 (9.8) months. There was no significant difference between the 2 groups (*P* = .480). In addition, there was no significant difference in curative resection rate across the GI endoscopists (*P* = .778).

## 4 Discussion

Of the 85 papillary adenocarcinoma-type EGC patients, 54 underwent surgery and 33 underwent ESD. LN metastasis was observed in 25.0% (n = 13) of the patients who underwent surgery. Of the 33 patients who underwent ESD, 21 (63.6%) exhibited absolute indications; 12 (36.4%) exhibited expanded indications, and no patients exhibited beyond expanded indication. LN metastasis was not observed in 3 patients who underwent surgery after ESD. The overall LN metastasis rate of papillary adenocarcinoma-type EGC was 23.6% (13/55), which included 3 patients who underwent surgery after ESD. The LN metastasis rate was 5.6% (1/18) in mucosal cancer and 32.4% (12/37) in submucosal cancer. According to previous studies, the LN metastasis rate increases in EGC involving the submucosa or undifferentiated-type EGC. One study indicated that the LN metastasis rate was 15.0% in differentiated-type EGC involving the submucosa whose lesions were smaller than 3 cm. Moreover, the LN metastasis rate was 1.7% for differentiated-type EGC with lesions smaller than 2 cm.^[12,13]^ The LN metastasis rate of undifferentiated-type EGC is relatively high, being 4.2% to 4.9% in mucosal cancer and 19.0% to 23.8% in submucosal cancer.^[14–16]^ In a study of papillary adenocarcinoma-type EGC, the LN metastasis rate was 7.1% and 22.9% in mucosal and submucosal cancer, respectively, which is similar to what we observed.^[17]^ Eventually, it is assumed that the LN metastasis rate of papillary adenocarcinoma-type EGC may be higher than that of differentiated-type EGC and similar to that of undifferentiated-type EGC; however, additional study is needed owing to the small sample size.

It is known that papillary adenocarcinoma accounts for 6% to 11% of all gastric cancer types and for less than 1% of EGC.^[8]^ As it is a rare disease, only a few studies have focused on papillary adenocarcinoma and hence, little is known about its characteristics or prognosis. When compared to other gastric cancer types, it was more often found in the proximal organs; exhibiting a macroscopic elevated type; and underwent extensive LN, peritoneal, and hepatic metastasis during the early stage; hence, its prognosis was poor.^[7,11]^ In a study of 6276 gastric cancer patients, the LN metastasis rate was the highest in papillary adenocarcinoma of other histologic types. Moreover, extensive liver and peritoneal metastasis were documented and the 10-year survival rate was quite low.^[11]^ In this study, the incidence rate of papillary adenocarcinoma-type EGC was 4.5% (85/1879). Most lesions were located in the lower third of the stomach and exhibited a macroscopically elevated type. Although the authors only considered cases of EGC, LN metastasis was observed in 23.6% of cases, and during the observation period, liver metastasis was observed in 2 patients, peritoneal metastasis was observed in 1 patient, and 3 patients died. Since papillary adenocarcinoma shows a high LN metastasis rate even in an early stage. Therefore, it is necessary to consider the risk of LN metastasis during treatment.

Papillary adenocarcinoma is categorized as a differentiated-type adenocarcinoma under the Japanese classification;^[10]^ however, clinicians continue to debate on whether ESD indications can be applied to the existing differentiated-type EGC. In order to evaluate the application of existing ESD indications in the treatment of papillary adenocarcinoma-type EGC, we compared the clinical outcomes of patients who underwent ESD to treat papillary adenocarcinoma-type EGC to those of 926 patients who underwent ESD to treat well- to moderately-differentiated adenocarcinoma-type EGC. Between the 2 groups, there were no significant differences in baseline characteristics, including gender, age, macroscopic type, tumor location, depth of invasion, and tumor size. In addition, there were no differences in clinical outcomes, including LVI, resection margin tumor invasion, procedure-related complication, *en bloc* resection, and curative resection rate, nor in long-term outcomes, including local recurrence and overall survival. Consequently, when compared to the existing ESD indication group, papillary adenocarcinoma-type EGC exhibited comparable short- and long-term clinical outcomes after ESD. It is known that additional surgery after ESD is required in approximately 2.1% to 14.6% of cases.^[18,19]^ In this study, 9.1% (n = 3) of patients who were diagnosed with papillary adenocarcinoma-type EGC and underwent ESD required additional surgery, which is similar to previously reported rates.

ESD can improve a patient's quality of life, reduce surgery-associated morbidity, and can preserve the stomach; however, it is necessary to consider the risk of LN metastasis.^[20,21]^ In a study that analyzed the risk factors of LN metastasis in 49 patients who underwent surgery with papillary adenocarcinoma-type EGC, LVI was the only risk factor (*P* = .016).^[17]^ Similarly, we found that patients who experienced LN metastasis tended to exhibit higher submucosal invasion (*P* = .039) and LVI (*P* = .002) than did patients who did not develop such a complication. However, our multivariate analysis indicated that only LVI was an independent risk factor for LN metastasis. In order to determine whether ESD is suitable for treating papillary adenocarcinoma-type EGC, it is necessary to consider the risk of LN metastasis.

This study had several limitations. First, since this was a single center-retrospective study, a selection bias may have existed. However, since our hospital is the only tertiary medical center in the area, various patients visit from neighboring areas and so the effect of this bias was most likely minimal. Second, since papillary adenocarcinoma is a rare disease, the sample size was relatively small and so it is difficult to generalize and apply the results of this study. Notably, this is the first study that compared the clinical outcomes after ESD in the papillary adenocarcinoma-type EGC group and the group of patients with existing ESD indications.

In conclusion, LVI is an independent risk factor for LN metastasis in papillary adenocarcinoma-type EGC. In addition, when compared with the existing ESD indication group, ESD in papillary adenocarcinoma-type EGC similarly showed acceptable clinical outcomes. However, given that the risk of LN metastasis is relatively higher in papillary adenocarcinoma-type EGC than in differentiated-type EGC, ESD should be performed in such patients only after carefully evaluating the associated risk of LN metastasis. Ultimately, an additional, large-scaled, prospective study is required to further elucidate the risks of LN metastasis and support clinicians in evaluating the suitability of ESD in papillary adenocarcinoma-type EGC.

## Author contributions

**Conceptualization:** Ju Seok Kim, Sun Hyung Kang, Hee Seok Moon, Jae Kyu Sung, Hyun Yong Jeong.

**Data curation:** Jae Ho Park.

**Formal analysis:** Jae Ho Park.

**Methodology:** Jae Ho Park, Ju Seok Kim, Sun Hyung Kang.

**Project administration:** Ju Seok Kim, Hyun Yong Jeong.

**Software:** Jae Kyu Sung.

**Supervision:** Ju Seok Kim, Sun Hyung Kang, Jae Kyu Sung, Hyun Yong Jeong.

**Validation:** Jae Ho Park, Ju Seok Kim, Hyun Yong Jeong.

**Visualization:** Ju Seok Kim, Hee Seok Moon.

**Writing – original draft:** Jae Ho Park.

**Writing – review & editing:** Hee Seok Moon.

Ju Seok Kim orcid: 0000-0002-6190-6506.
